# *De Novo ZNF292* Variants Cause Neurodevelopmental Disorder with Short Stature: A Clinical Case Series of Eight Individuals

**DOI:** 10.3390/genes17080950

**Published:** 2026-08-13

**Authors:** Yaping Shen, Rongrong Pan, Chen Liu, Jing Zheng, Xin Yang

**Affiliations:** Department of Genetics and Metabolism, Children’s Hospital, Zhejiang University School of Medicine, National Clinical Research Center for Children and Adolescents’ Health and Disease, Hangzhou 310052, China

**Keywords:** *ZNF292* gene, intellectual developmental disorder, short stature

## Abstract

**Background**: Pathogenic variants in *ZNF292* cause intellectual disability type 64 (MIM#619188), characterized by intellectual impairment ranging from mild to severe, speech delay, and autism spectrum disorder. However, reports of *ZNF292*-related neurodevelopmental disorders remain scarce, and most published studies are based on multi-center cohorts. **Methods**: We performed whole-exome sequencing in eight unrelated individuals presenting with unexplained neurodevelopmental disorders, including global developmental delay and/or intellectual disability. Detailed clinical characterization, neuroimaging, and developmental assessments were conducted. **Results**: Seven *de novo* variants in *ZNF292* were identified, including two nonsense and five frameshift variants, namely, c.4189C>T (p.Arg1397Ter), c.6343C>T (p.Arg2115Ter), c.1533del (p.Ile511Metfs*11)*,* c.3094dup (p.Ser1032Phefs*18), c.3997_3998del (p.Thr1333Glnfs*8), c.6028_6031del (p.Ala2010Ter) and c.6160_6161del (p.Glu2054Lysfs*14). Among these, c.1533del (p.Ile511Metfs11)*,* c.3094dup (p.Ser1032Phefs18) and c.3997_3998del (p.Thr1333Glnfs*8) are reported here for the first time. All variants were classified as pathogenic. All individuals exhibited global developmental delay, intellectual disability, and short stature. The majority presented with language impairment, motor delays, autism spectrum features, and dysmorphic facial features, while brain magnetic resonance imaging revealed nonspecific abnormalities such as ventriculomegaly. **Conclusions**: This study contributes additional cases to the expanding phenotypic and mutational spectrum of *ZNF292*-related neurodevelopmental disorder. Growth retardation was observed in all eight individuals, but given the limitations of a single-center referral cohort, this observation should be interpreted with caution and requires validation in larger studies.

## 1. Introduction

The *ZNF292* gene, located on chromosome 6q14.3, encodes a highly conserved C2H2-type zinc finger transcription factor containing 16 zinc finger domains, two nuclear localization signals, and three coiled-coil domains [1]. As a sequence-specific DNA-binding protein, ZNF292 recognizes and binds to the promoters of neuronal and synaptic genes, thereby directly regulating the expression of multiple neurodevelopmental disorder risk genes [2]. Genome-wide occupancy analyses in human neural progenitor cells have revealed that ZNF292-associated binding sites are significantly enriched for motifs recognized by bHLH, homeodomain, and Ets family transcription factors, suggesting that ZNF292 participates in cooperative transcriptional networks during neurodevelopment [2].

ZNF292 is among the most highly expressed zinc finger proteins in the developing human brain, with peak expression occurring during the prenatal period, particularly in the cerebellum and developing cortex [1]. Recent functional studies utilizing human stem cell models have demonstrated that ZNF292 is essential for cortical interneuron development. ZNF292 deficiency leads to premature neuronal differentiation coupled with impaired maturation, resulting in deficient interneurons with altered channel activities, elevated GABA responsiveness, and hallmarks of neuronal hyperactivity [2]. These findings establish ZNF292 as a critical regulator of transcriptional programs governing neuronal lineage commitment, synaptogenesis, and cortical circuitry formation.

In 2020, Mirzaa et al. identified 24 heterozygous nonsense or frameshift variants in *ZNF292* in 28 unrelated patients with developmental delay from multiple centers, and formally defined this autosomal dominant condition as intellectual developmental disorder-64 (MRD64; MIM#619188). MRD64 is characterized primarily by mild to severe intellectual disability, speech delay and autism spectrum disorder. Additional variable features include tone abnormalities, dysmorphic features, growth failure, brain MRI abnormalities, ocular features, feeding issues, constipation, microcephaly, skeletal abnormalities and epilepsy [1]. To date, reports on *ZNF292* remain limited, and most available data have been derived from multi-center studies. Notably, growth retardation has been reported only as an inconsistent finding in small subsets of patients.

In this study, whole-exome sequencing identified seven *ZNF292* variants in eight unrelated patients from a single center, three of which were previously unreported. While cognitive and language delays are well-established core features, growth retardation was observed in all eight individuals, suggesting that short stature may represent a more consistent component of the *ZNF292* phenotype than previously recognized. Our findings contribute additional cases that further delineate the clinical and mutational spectrum of this emerging neurodevelopmental syndrome.

## 2. Materials and Methods

### 2.1. Patients

Eight unrelated individuals with unexplained neurodevelopmental disorders were recruited from Children’s Hospital, Zhejiang University School of Medicine between 2021 and 2024. Inclusion criteria were (1) global developmental delay and/or intellectual disability; (2) absence of acquired neurological injury; and (3) availability of parental DNA for trio-based analysis.

Clinical data were systematically collected, including prenatal and perinatal history, developmental milestones, neurological examination, behavioral assessment, and neuroimaging findings. Written informed consent was obtained from the parents or legal guardians. The study involving human participants were reviewed and Approved by the Ethics Committee of Children’s Hospital, Zhejiang University School of Medicine (2024-IRB-0147-P-01).

### 2.2. Whole-Exome Sequencing

Whole-exome sequencing was performed on genomic DNA from probands and unaffected parents. Exome capture was conducted using the KAPA HyperExome V2 Probes kit (Roche Molecular Systems, Inc., Pleasanton, CA, USA), followed by paired-end 150-bp sequencing on the MGI DNBSEQ-T7 platform (MGI Tech., Beijing, China).

### 2.3. Bioinformatics Analysis

Raw reads were aligned to the GRCh37/hg19 reference genome using BWA-MEM (v2.2.1). Duplicate reads were marked with Picard (v2.27), and variants were called using GATK HaplotypeCaller (v4.0) following best-practice guidelines. Variants were retained only if they met the following criteria: QUAL ≥ 50, GQ ≥ 20, DP ≥ 10, VAF ≥ 20% for heterozygous variants, call rate ≥ 90% across all samples, and exclusion of variants in low-complexity regions and segmental duplications. Rare variants were filtered against gnomAD (AF < 0.1%). Copy-number analysis was performed using CNVkit (v0.9.9); segments with log_2_ ratios > 0.58 or <−1.0 were visually inspected in IGV. Mosaic variants were screened at relaxed VAF thresholds (≥5%) in NDD-associated genes.

Candidate variants were systematically prioritized under three inheritance models: (1) coding *de novo* SNVs and indels in known NDD/growth-disorder genes; (2) compound-heterozygous or homozygous rare variants in autosomal recessive NDD genes (gnomAD AF < 1%); and (3) hemizygous rare variants on the X chromosome in known X-linked intellectual disability genes in male probands. Variant annotation was performed with VEP (v113) and ANNOVAR (v20250302) with reference to standard population and disease databases (gnomAD, dbSNP, OMIM, HGMD, and ClinVar). The functional impact of candidate variants was predicted using established in silico tools (SIFT (v6.2.1), PolyPhen-2 (v2.2.3), CADD (v1.6), and REVEL). All variants were classified according to the ACMG/AMP guidelines [3].

Maternity and paternity were confirmed by SNP-based identity-by-descent (IBD) analysis to exclude sample mix-up or non-biological parentage. Candidate pathogenic variants were subsequently validated by bidirectional Sanger sequencing of the proband and both parents to confirm the variant and verify its *de novo* status. Quality control metrics are summarized in Table S1. Detailed information on the databases and in silico tools is provided in Table S2.

### 2.4. Clinical and Developmental Evaluation

Developmental assessment included gross motor, fine motor, language, and social domains. Intellectual disability severity was categorized as mild, moderate, or severe based on standardized developmental scales. Autism spectrum disorder features were evaluated according to DSM-5 criteria. Behavioral abnormalities, including hyperactivity, attention deficit, and anxiety, were systematically recorded.

### 2.5. MRI Examination

The MRI examination was performed on a Siemens 1.5T MRI system, with the imaging protocol comprising axial T1WI, axial T2WI, axial FLAIR, and sagittal T1WI.

## 3. Results

### 3.1. Clinical Characteristics

Detailed clinical data were collected from eight affected individuals in eight unrelated families. The cohort comprised five males and three females, all from healthy, non-consanguineous families. Ages at initial clinical evaluation ranged from 2 to 13 years. All individuals exhibited a consistent core phenotype comprising global developmental delay, intellectual disability, and short stature. Despite this shared core, the cohort displayed marked phenotypic heterogeneity. The severity of intellectual disability ranged from mild to severe, and the pattern of growth restriction varied, with some individuals showing reduced birth parameters and others demonstrating progressive postnatal growth failure. Language impairment was observed in six patients (75%), including one individual with complete absence of expressive language, whereas hypotonia were noted in five (62.5%). Dysmorphic facial features were present in four patients (50%), with variable manifestations including blepharophimosis, hypertelorism, low-set ears, and bulbous nasal tip. Nonspecific abnormalities on brain magnetic resonance imaging (MRI) were identified in five patients (62.5%), such as ventriculomegaly or widened subarachnoid spaces. However, the remainder had normal neuroimaging. Certain features were restricted to individual cases, notably congenital heart disease requiring surgical correction and feeding difficulties. Skeletal abnormalities were absent across the cohort. The detailed clinical features in our cohort are summarized in Table 1. The clinical characteristics of all *ZNF292* positive patients are compared in Table S3.

### 3.2. Molecular Findings

**Figure 1:**
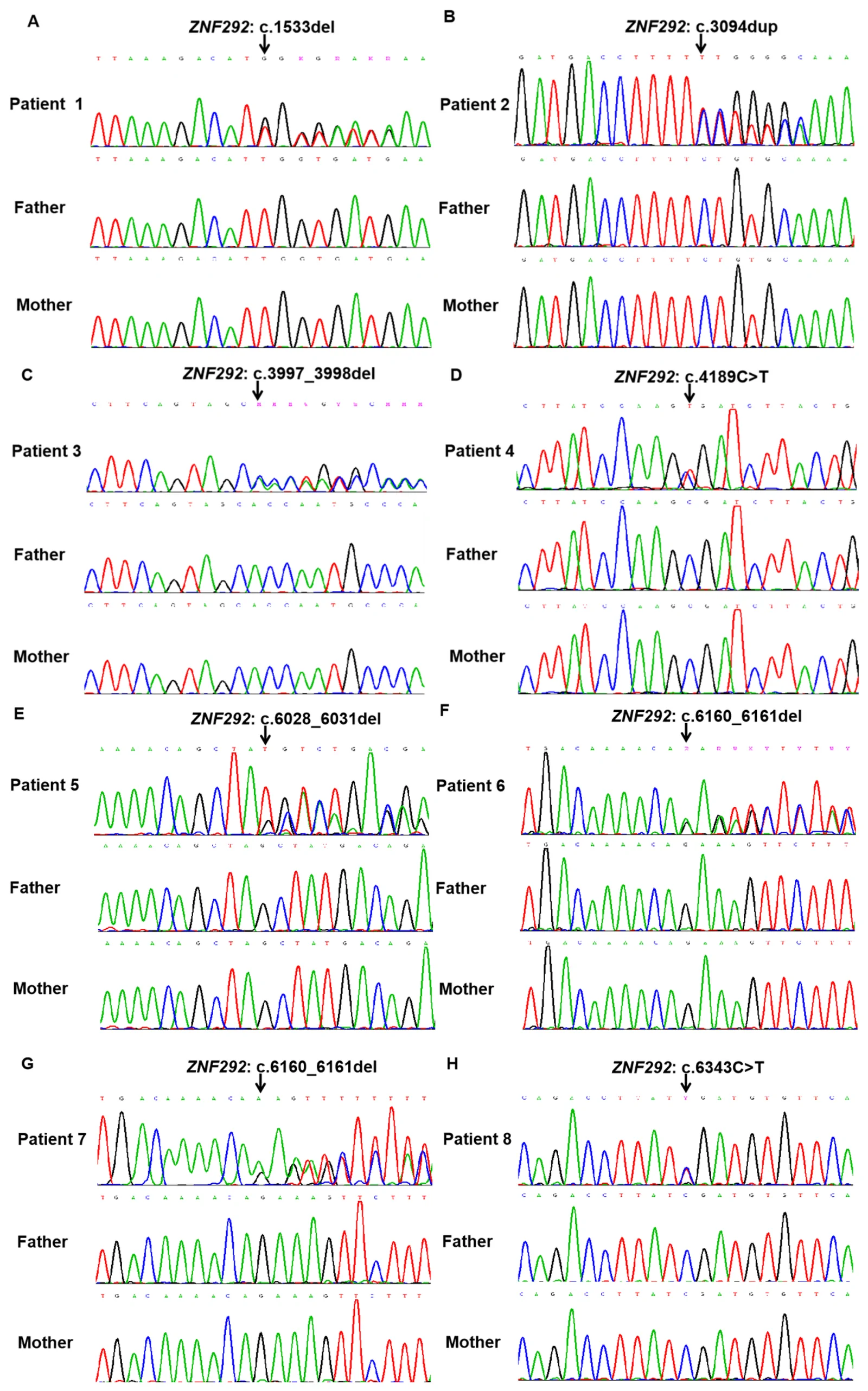
Sanger sequencing chromatograms of probands and parents confirming the *de novo* origin of *ZNF292* variants (NM_015021.3). (**A**) Patient 1: c.1533del (p.Ile511Metfs*11); (**B**) Patient 2: c.3094dup (p.Ser1032Phefs*18); (**C**) Patient 3: c.3997_3998del (p.Thr1333Glnfs*8); (**D**) Patient 4: c.4189C>T (p.Arg1397Ter); (**E**) Patient 5: c.6028_6031del (p.Ala2010Ter); (**F**) Patient 6: c.6160_6161del (p.Glu2054Lysfs*14); (**G**) Patient 7: c.6160_6161del (p.Glu2054Lysfs*14); (**H**) Patient 8: c.6343C>T (p.Arg2115Ter). The black arrows indicate the variant positions. The chromatograms demonstrate that all variants are absent in all parents.

Whole-exome sequencing identified seven *ZNF292* variants in eight patients from eight unrelated families, namely, NM_015021.3: c.4189C>T (p.Arg1397Ter), c.6343C>T (p.Arg2115Ter), c.1533del (p.Ile511Metfs*11), c.3094dup (p.Ser1032Phefs*18), c.3997_3998del (p.Thr1333Glnfs*8), c.6028_6031del (p.Ala2010Ter) and c.6160_6161del (p.Glu2054Lysfs*14). No other variants or copy number variations can explain the observed phenotype. Three of these variants, c.1533del (p.Ile511Metfs*11), c.3094dup (p.Ser1032Phefs*18) and c.3997_3998del (p.Thr1333Glnfs*8) have not been previously reported in the literature or public databases. Families 6 and 7 carried the same variant, c.6160_6161del (Glu2054Lysfs*14). All variants were detected in the probands but were absent in both parents, confirming their *de novo* origin (Figure 1). All variants were classified as pathogenic (Table 2).

## 4. Discussion

Heterozygous *ZNF292* variants, whether *de novo* or inherited in a dominant manner, have been linked to a range of neurodevelopmental features, including intellectual disability (ID) and autism spectrum disorder (ASD) [1]. In 2012, Joep et al. first reported a patient with intellectual disability harboring a *de novo* c.2828_2829del(p.Leu943Glnfs*5) variant in *ZNF292* [6]. Subsequently, several additional patients with autism spectrum disorder or broader neurodevelopmental disorders carrying truncating variants in *ZNF292* were reported [7,8,9]. In 2020, Mirzaa et al. reported 24 heterozygous nonsense or frameshift variants in *ZNF292* in 28 unrelated patients with developmental delay from multiple centers; the predominant clinical phenotype was intellectual disability of variable severity, with or without ASD [1].

The core clinical phenotype observed in our cohort aligns with the established spectrum of *ZNF292*-related disorder. All eight individuals exhibited intellectual disability of variable severity, consistent with the high prevalence (96%) reported by Mirzaa et al. [1]. Speech and language impairment was prominent in six patients (75%), comparable to the 93% prevalence previously reported [1], though the severity ranged from mild delay to complete absence of expressive language. Notably, one patient showed no apparent language deficit, highlighting the phenotypic variability of this condition. Motor manifestations were also common; hypotonia was the most frequent motor sign, present in four individuals. Autism spectrum disorder-related features, reported in 55–70% of patients in previous cohorts [1,7,9], were observed in four of our patients (50%). Overall, the neurodevelopmental profile in our series is consistent with prior multi-center studies, supporting *ZNF292* as a recurrent cause of syndromic intellectual disability.

Growth impairment was observed in all eight individuals (100%) in our cohort, with patterns varying from reduced birth parameters to progressive postnatal growth restriction. This frequency exceeds the 39% (11/27) previously reported by Mirzaa et al. [1]. However, this observation should be interpreted with caution. All *ZNF292*-positive individuals identified during the study period were enrolled irrespective of growth status, and short stature was not a criterion for referral or genetic testing. The higher observed prevalence likely reflects systematic growth assessment as part of the standardized clinical evaluation protocol at our referral center, and confirmation in larger, unselected cohorts is warranted before any inference about enrichment can be made. Interestingly, Li et al. (2025) also reported a patient with a *ZNF292* truncating variant and marked short stature (−3.75 SD) [10], suggesting that growth retardation may represent a variable feature within the phenotypic spectrum of this disorder.

Beyond the core neurodevelopmental and growth phenotypes, our cohort exhibited marked clinical heterogeneity. Dysmorphic facial features were present in four patients (50%), with variable manifestations including blepharophimosis, hypertelorism, low-set ears, and bulbous nasal tip. Nonspecific brain MRI abnormalities were identified in five patients (62.5%), such as ventriculomegaly or widened subarachnoid spaces, whereas the remainder had normal neuroimaging. Additional features were restricted to individual cases, including congenital heart disease requiring surgical correction and feeding difficulties. Skeletal abnormalities were absent across the cohort. Clinical features and their frequencies in all reported individuals with *ZNF292* variants are summarized in Table S4.

**Figure 2:**
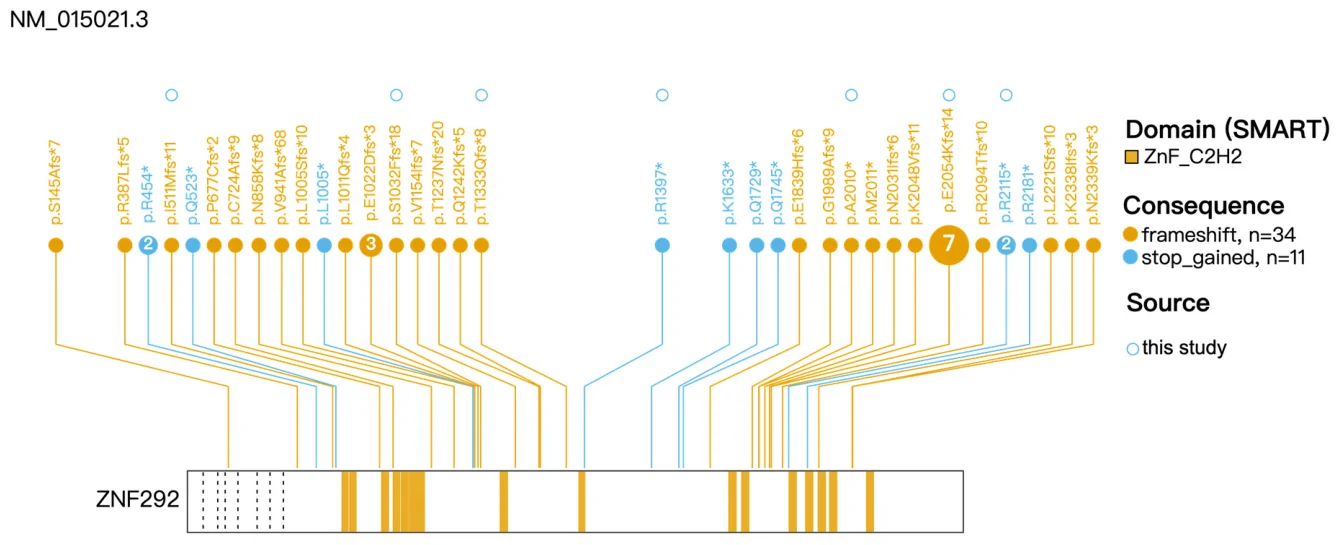
Distribution of pathogenic and likely pathogenic variants in *ZNF292*.

By June 2025, HGMD (Professional 2025Q2) had documented 31 pathogenic *ZNF292* variants associated with neurological disease, all of which were truncating changes [1,11,12,13,14,15,16,17] (Figure 2). The majority of reported variants (30/31) were located in exon 8 of *ZNF292*, with only a single variant, c.433del (p.Ser145Alafs*7), identified in exon 4 (Figure 2). This distribution likely reflects the structure of the gene: *ZNF292* comprises eight exons, and exon 8 alone encodes approximately 85% of the protein, making the observed variant distribution consistent with relative coding length rather than biological clustering. In our study, we identified seven *ZNF292* variants in eight patients from eight unrelated families. There were two heterozygous nonsense variants-c.4189C>T (p.Arg1397Ter) and c.6343C>T (p.Arg2115Ter); five frameshift variants-c.1533del (p.Ile511Metfs*11), c.3094dup (p.Ser1032Phefs*18), c.3997_3998del (p.Thr1333Glnfs*8), c.6028_6031del (p.Ala2010Ter) and c.6160_6161del (p.Glu2054Lysfs*14). All variants were localized to exon 8. The three novel variants reported herein (c.1533del, c.3094dup and c.3997_3998del) further expand the known mutational repertoire. Notably, the variant c.6160_6161del (p.Glu2054Lysfs*14) was observed in two unrelated families in our cohort and has now been reported in a total of seven individuals [1,18], making it the most frequently observed pathogenic variant in *ZNF292* to date. The clinical presentations among carriers of this recurrent variant range from severe global developmental delay to mild neurodevelopmental impairment, further illustrating the variable expressivity of this disorder.

Beyond its established role in neurodevelopment, *ZNF292* has also been implicated as a tumor suppressor gene. Somatic frameshift variants have been identified in MSI-H gastric (8.8%) and colorectal (13.9%) cancers [19], and it has been proposed as a two-hit target in prostate cancer [20] and chronic lymphocytic leukemia [21], highlighting its pleiotropic roles across diverse tissues. The pathogenic variants in our cohort are germline alterations associated with constitutional developmental phenotypes, distinct from the somatic events implicated in tumorigenesis. While no malignancies were observed in our pediatric cohort, long-term oncological surveillance may be prudent as these individuals transition into adulthood.

In addition, this study has several limitations. First, as a single-center case series with a small sample size, our findings may be subject to ascertainment bias and require validation in larger, multi-center cohorts. Second, the absence of functional experimental data precludes definitive conclusions regarding the molecular mechanism by which the identified variants contribute to the clinical phenotype. Third, the high prevalence of growth retardation in our cohort may reflect systematic growth assessment at our referral center rather than true enrichment. Longitudinal follow-up and systematic growth evaluation in future cohorts will be necessary to clarify the prevalence and natural history of growth impairment in *ZNF292*-related disorder.

In conclusion, this study contributes additional cases to the expanding phenotypic and mutational spectrum of *ZNF292*-related neurodevelopmental disorder. The identification of three novel variants and the detailed clinical characterization of eight patients provide valuable data for clinical recognition and genetic counseling. Future studies incorporating functional validation and larger, systematically phenotyped cohorts will be essential to elucidate the full clinical spectrum and underlying pathogenic mechanisms of this condition.

Figure 2 is a schematic representation of pathogenic and likely pathogenic *ZNF292* variants reported in the literature together with those identified in the present study. Variants are mapped according to their corresponding amino acid positions in the ZNF292 protein. The asterisk (*) indilcted stop codon. Blue dots indicate stop_gained variants, whereas orange dots represent frameshift variants. The blue hollow ring represent variants identified in our cohort. The size of each circle is proportional to the number of reported individuals carrying the corresponding variant, with the number shown inside circles for recurrent variants. All 16 C2H2-type zinc finger domains are encoded within exon 8, which comprises approximately 85% of the total coding sequence; consequently, the observed predominance of variants within this exon is presented as a descriptive observation and does not imply statistically significant biological clustering. Detailed information on zinc finger domain positions and all variants shown (including standardized HGVS nomenclature, inheritance pattern, ACMG classification, and the number of zinc fingers lost or truncated) is provided in Tables S5 and S6.

## Figures and Tables

**Table 1:** Summary of the clinical features in this cohort (N = 8).

Patient No.	P1	P2	P3	P4	P5	P6	P7	P8
cDNA change	c.1533del	c.3094dup	c.3997_3998del	c.4189C>T	c.6028_6031del	c.6160_6161del	c.6160_6161del	c.6343C>T
Predicted protein alteration	p.Ile511Metfs*11	p.Ser1032Phefs*18	p.Thr1333Glnfs*8	p.Arg1397*	p.Ala2010*	p.Glu2054Lysfs*14	p.Glu2054Lysfs*14	p.Arg2115*
Exon	8	8	8	8	8	8	8	8
Inheritance	*de novo*	*de novo*	*de novo*	*de novo*	*de novo*	*de novo*	*de novo*	*de novo*
Gestational age (weeks)	38 W	38 W	37 W	40 W	38 W	38 W	39 W	35 W
Birth weight (grams)	2700 g	2900 g	1850 g	3350 g	3000 g	2900 g	3150 g	2350 g
Birth length (cm)	48 cm	50 cm	40 cm	50 cm	50 cm	50 cm	50 cm	NA
Gender	M	F	M	M	F	M	M	F
Assessed age	9Y	12Y8M	6M	2Y8M	2Y2M	1Y5M	6Y	3Y1M
Assessed Height	123.5 cm (-2SD)	140.0 cm (-2SD)	60.0 cm (<P3)	90.0 cm (-2SD)	78.0 cm (<P3)	71.1 cm (<P3)	105.0 cm (<P3)	89.0 cm (<P3)
Assessed Weight	18.0 kg	39.0 kg	5.7 kg	15.0 kg	8.4 kg	7.1 kg	14.5 kg	11.5 kg
ID	Mild ID	Mod ID	Mod ID	Severe ID	Mod ID	Mod ID	Mild ID	Mild ID
ASD	+	+	+	+	–	–	–	–
behavior abnormalities	ADHD	ADHD	ADHD	ADHD	NA	–	–	–
Speech delays	–	+	–	speech absent	+	+	+	+
Tone abnormalities	–	hypotonia	NA	hypotonia	NA	hypotonia	hypotonia	hypotonia
Feeding difficulties	–	–	–	–	–	+	–	–
Skeletal abnormalities	–	–	–	–	–	–	–	–
Characteristic facial features	–	Blepharophimosis, hypertelorism, and depressed nasal bridge	Blepharophimosis and low-set ears	Bulbous nasal tip, low-set ears, Elongated philtrum, high-arched palate, ligamentous laxity	–	Prominent ears, bulbous nasal tip	–	–
Cardiac issues	–	Congenital heart disease surgery (17M)	–	–	–	–	–	–
MRI findings	Enlarged occipital cistern, possible arachnoid cyst, increased shadows around bilateral frontal and parietal lobe blood vessels	Bilateral lateral ventricles slightly plump in shape	The extracranial space and sulcus are wide, and the body of both lateral ventricles is enlarged	Normal	ND	ND	Bilateral lateral ventricles dilated, with multiple small patchy abnormal signals near the bilateral lateral ventricles	Widening of perivascular spaces in bilateral ventricular trigone and bilateral semiovale centers
Other	–	–	Widely spaced nipples, brachydactyly with small hands and feet, dorsal hand/foot puffiness, flexion of the 2nd and 5th fingers, and adducted thumbs	–	–	–	–	–
Reported	Not	Not	Not	[4]	[5]	[1]	[1]	[1]

Notes: *ZNF292* reference sequence (NM_015021.3). Abbreviations: ADHD, attention-deficit hyperactivity disorder; ASD, autism spectrum disorder; F, female; ID, intellectual disability; M, male; NA, not applicable; ND, no data; *, stop codon.

**Table 2:** *ZNF292* variants identified in eight patients of this study.

Patient No.	Position (hg19)	Position (hg38)	Nucleotide Change (NM_015021.3)	Amino Acid Alteration (NP_055836.1)	Zygosity	Inheritance	Variants Type	VAF	GnomAD MAF	CADD	phastCons	ACMG Classification	Reported
P1	chr6: 87964878	chr6: 87255160	c.1533del	p.(Ile511Metfs*11)	het	DN	frameshift	48%	0	NA	NA	P: PVS1 +PS2 + PM2	Not
P2	chr6: 87966437	chr6: 87256719	c.3094dup	p.(Ser1032Phefs*18)	het	DN	frameshift	52%	0	NA	NA	P: PVS1 +PS2 + PM2	Not
P3	chr6: 87967342	chr6: 87257624	c.3997_3998del	p.(Thr1333Glnfs*8)	het	DN	frameshift	47%	0	NA	NA	P: PVS1 +PS2 + PM2	Not
P4	chr6: 87967536	chr6: 87257818	c.4189C>T	p.Arg1397Ter	het	DN	nonsense	53%	0	35	1	P: PVS1 +PS2 + PM2	[4]
P5	chr6: 87969369	chr6: 87259651	c.6028_6031del	p.(Ala2010Ter)	het	DN	frameshift	51%	0	NA	NA	P: PVS1 +PS2 + PM2	[5]
P6	chr6: 87969505	chr6: 87259787	c.6160_6161del	p.(Glu2054Lysfs*14)	het	DN	frameshift	48%	0	NA	NA	P: PVS1 +PS2 + PM2	[1]
P7	chr6: 87969505	chr6: 87259787	c.6160_6161del	p.(Glu2054Lysfs*14)	het	DN	frameshift	54%	0	NA	NA	P: PVS1 +PS2 + PM2	[1]
P8	chr6: 87969690	chr6: 87259972	c.6343C>T	p.(Arg2115Ter)	het	DN	nonsense	49%	0	39	0.993	P: PVS1 +PS2 + PM2	[1]

*: stop codon; DN: *de novo*; Het: heterozygous mutation; NA: not applicable; P: pathogenic.

## Data Availability

The data generated in this study can be found within the article. Raw data are available from the corresponding author on request.

## Supplementary Materials

The following supporting information can be downloaded at: https://www.mdpi.com/article/10.3390/genes17080950/s1, Table S1: The information of mean sequencing depth and target region coverage; Table S2: The information of software in silico prediction algorithms and database; Table S3: The clinical characteristics of the current patients and previously reported ZNF292-postive individuals (N = 37); Table S4: The summary of the clinical features of *ZNF292* mutation-positive individuals (N = 37); Table S5: The ZnF_C2H2 position of *ZNF292*; Table S6: The information of the variants in Figure 2.
